# Tannic Acid : Its Internal Administration for Hemorrhage after Tooth Extraction

**Published:** 1890-08

**Authors:** W. L. Roberts

**Affiliations:** WEYMOUTH, MASS.


ARTICLE IV.

### TANNIC ACID: ITS INTERNAL ADMINISTRATION FOR HEMORRHAGE AFTER TOOTH EXTRACTION.

BY DR. W. L. ROBERTS, WEYMOUTH, MASS.

Read before the Union Meeting, Springfield, Mass., October 25, 1889.

Tannic acid, as we all know, has a yellowish-white color and strongly astringent taste. It is decomposed and entirely dissipated when thrown on red-hot iron. It is very soluble in water and less so in alcohol and ether. Its solution reddens litmus and produces with solution of gelatine a white flocculent precipitate, and with solution of the alkaloids white precipitates, and is very soluable in acetic acid. Dose from three to ten grains.

Very little has ever been written, and less said, upon the internal administration of this most valuable adjunct to our list of hæmostatics.

The most of us do at times have those perplexing cases of hemorrhage from extraction which are very hard to control, and necessarily resort to numerous devices to bring about the desired results, all or a portion of which are very disagreeable to both dentist and patient.

Tannic acid, administered internally in proper doses, will stop, I believe, any case of hemorrhage caused by tooth extraction, in from thirty minutes' to one and one-half hours' time. The manner and results of administering this very simple remedy I will illustrate by one case in practice.

I was called, March 25 1885 at 8 P. M., to check hemorrhage from the lower gum of a lady, caused by the removal of eight badly-decayed and broken-down teeth. They were removed while patient was under nitrous oxide gas, with no more laceration of the gums than generally occurs. Patient did not bleed very profusely at the time but as she was a hemorrhagic diathesis I kept her there until it had entirely ceased, with instructions to call me at once if there was a return of the hemorrhage to any great extent. As she lived some distance away, and being at home alone, I did not hear from her until about 7 P. M., when her husband came to me with the information that his wife was bleeding to death. I immediately went to their residence and found the patient in a bad state indeed. Pulse was very weak, and she looked about ready to expire, but there was life, and, upon inquiry, I found that she had expectorated nearly one quart of blood. Upon examination I found blood oozing from all portions of the gum. I immediately placed three grains of tannic acid in one third glass of water and gave her two teaspoonfuls every five minutes until she had taken three doses, then two teaspoonfuls every fifteen minutes; after the second dose the flow had diminished to such an extent that I left them with instructions to administer the same amount every half-hour which they did, and were only obliged to give two doses before it ceased entirely, with no return.

I have used tannic acid for the last five years, whenever occasion presented, with the same good results, in fact it has never failed me.

Now, what is its physiological action. From experiments on animals with very large doses, it appears that this acid renders the gastric mucous membrane pale and lustreless and coagulates its mucous. Injected into the blood-vessels it coagulates the albumen of the blood. To the former of these actions, as well as to its inherent astringency, must be attributed the dryness and sense of constriction which it produces in the mouth and fauces.

We are told that tannic acid is not absorbed and does not circulate as such, but is converted into gallic acid. It is true that in this form alone it is found in the blood and urine. Indeed, it could not remain in the blood without coagulating it, as experiments on rabbits demonstrate; but as gallic acid is not an astringent, it is difficult, while admitting the conversion of the one acid into the other, to explain the therapeutic operation of the latter.

Several theories have been proposed for this purpose, but, as far as I can find, all of them are unsatisfactory.

Lewin has shown that the coagula of albuminous substances formed by tannic acid, if made slightly neutral, loses its coagulating power. Thus an albuminate of tannin formed in the blood is dissolved again in an excess of that alkaline liquid. A small portion of tannin escapes neutralization and is discharged with the urine unchanged, and hence, according to Lewin, tannin, as such, may exert its power in all parts of the system. It would appear, also, that the hæmostatic and analogous qualities of tannic acid are due not to an action upon the blood, but upon the blood-vessels, by which they become contracted and the flow of blood through them is checked.

We also see in nervous patients frequent anomalies of the vaso-motor and trophic functions, but up to the present time we know comparatively little that is certain to the precise nature of their occurrence.

Physiology distinguishes two varieties of vaso motor nerves,—tbe vaso-constrictors and the vaso-dilators ; but since experiments have detected the latter variety in only a few places, for example, in the chorda tympani, the nervi erigentes, and the sciatic, they have not acquired a very great significance in human pathology. We are at present much more disposed to refer every abnormal constriction of the vessels to an irritation and every abnormal dilatation of the vessels to a paralysis, of the vaso-constrictor nerves, although perhaps pathological conditions of irritation of the vaso-dilators may not be at all rare. In regard to the anatomical course of the vaso-motor nerves, it is necessary to state that vaso-motor irritations may certainly proceed from the cerebrum, as is shown by the well known symptoms of blushing and pallor from mental emotions. In experiments on dogs Eulenburg and Landois have succeeded in producing a fall of temperature on the opposite side by irritating certain portions of the cortex in the immediate vicinity of the motor centres, and by extirpation of the same parts they have produced a rise in temperature. It is now known with certainty that there is an important vaso-motor centre in the medulla oblongata, the irritation of which, directly or reflexly, is followed by an almost universal vascular constriction, and its destruction by an almost universal vascular dilatation. We must probably seek the further course of the vaso-motor nerves largely in the lateral columns, from which they pass out chiefly by the anterior roots; but there are also experimental data suggesting the presence of vaso-motor nerves in the posterior roots. It is not known with certainty whether there is any decussation of the vaso-motor fibres, or, if there is, where it occurs. The larger part of the vaso-motor nerves collect, at any rate, in the principal trunks of the sympathetic, from which, as is well known, the separate plexuses that surround the vessels arise. It is probable, however, that there is in part a direct passage of vaso-motor fibres from the cord into the peripheral nerves. There are also ganglia in the walls of the blood-vessels themselves that are capable of maintaining the tone of the circulation in the absence of the central force or influence, but under ordinary conditions these minor ganglia are denominated and controlled by the one central power which unifies the whole system and renders it complete.

But, be it as it may, tannic acid, administered internally in proper doses, does stop the flow of blood from ruptured blood-vessels, and is a hæmostatic that I wish to commend to the careful consideration of all, for by looking deeper into this subject we shall all be benefited and perhaps add one more drug to our list.

Perhaps this paper does not present many new points or ideas to the older members especially, but my object is to revive, create, or bring to the front more modern ideas than our present materia medica furnishes. Our chosen profession is on the steady march upward in nearly all its branches, and may I not be pardoned if I ask, have we not neglected our pharmacopœa and turned our attention too much to the modern bridge-work or the Herbst method of tooth-filling? Is there any one department in dentistry that is so much neglected and yet be so thoroughly understood? Are we not falling behind just a little in this branch? Is there not a splendid opportunity for some of our eminent men to furnish us with an improved, concise, and modern materia medica?—*International Dental Journal.*

